# Kinematics, kinetics, and new insights from a contemporary analysis of the first experiments to produce cervical facet dislocations in the laboratory

**DOI:** 10.1002/jsp2.1336

**Published:** 2024-05-27

**Authors:** Ryan D. Quarrington, Robert Bauze, Claire F. Jones

**Affiliations:** ^1^ Adelaide Spinal Research Group, Centre for Orthopaedic & Trauma Research, Faculty of Health and Medical Sciences The University of Adelaide Adelaide South Australia Australia; ^2^ Adelaide Medical School The University of Adelaide Adelaide South Australia Australia; ^3^ School of Electrical and Mechanical Engineering The University of Adelaide Adelaide South Australia Australia; ^4^ Department of Orthopaedics and Trauma Royal Adelaide Hospital and The Queen Elizabeth Hospital Adelaide South Australia Australia; ^5^ Nuffield Department of Orthopaedic Surgery University of Oxford Oxford UK

**Keywords:** bilateral facet dislocation, injury, neck, spinal trauma

## Abstract

**Background:**

The first experimental study to produce cervical facet dislocation (CFD) in cadaver specimens captured the vertebral motions and axial forces that are important for understanding the injury mechanics. However, these data were not reported in the original manuscript, nor been presented in the limited subsequent studies of experimental CFD. Therefore, the aim of this study was to re‐examine the analog data from the first experimental study to determine the local and global spinal motions, and applied axial force, at and preceding CFD.

**Methods:**

In the original study, quasistatic axial loading was applied to 14 cervical spines by compressing them between two metal plates. Specimens were fixed caudally via a steel spindle positioned within the spinal canal and a bone pin through the inferior‐most vertebral body. Global rotation of the occiput was restricted but its anterior translation was unconstrained. The instant of CFD was identified on sagittal cineradiograph films (*N* = 10), from which global and intervertebral kinematics were also calculated. Corresponding axial force data (*N* = 6) were extracted, and peak force and force at the instant of injury were determined.

**Results:**

CFD occurred in eight specimens, with an intervertebral flexion angle of 34.8 ± 5.6 degrees, and a 3.1 ± 1.9 mm increase in anterior translation, at the injured level. For seven specimens, CFD was produced at the level of transition from upper neck lordosis to lower neck kyphosis. Five specimens with force data underwent CFD at 545 ± 147 N, preceded by a peak axial force (755 ± 233 N) that appeared to coincide with either fracture or soft tissue failure.

**Conclusions:**

Re‐examining this rich dataset has provided quantitative evidence that small axial compression forces, combined with anterior eccentricity and upper neck extension, can cause flexion and shear in the lower neck, leading to soft tissue rupture and CFD.

## Graphical abstract

Despite several decades of experimental cadaver work, the mechanisms underlying subaxial cervical facet dislocation (CFD) remain unclear. We re‐examined the analog data from the first experimental study (and one of only two to date) to produce CFD in full cervical spines (*n* = 8), and determined the local and global spinal motions, and applied axial force, that occurred during the injury event. The results of this contemporary analysis demonstrate empirically that small axial compression forces (545 ± 147 N), combined with anterior eccentricity of the head and upper neck extension, can cause supraphysiologic flexion and shear in the lower neck that produce CFD.

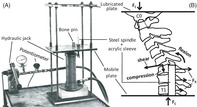

## 1 INTRODUCTION

Subaxial cervical facet dislocation (CFD) is a devastating consequence of cervical spine trauma and is associated with a spinal cord injury (SCI) rate of up to 87%.^1^ The injury can occur uni‐ or bilaterally, and frequently results from traffic and sports accidents, and falls,^1^ during which the external loading applied to the head and neck can be complex and variable. Concomitant fracture of the posterior elements, including the facets, often accompanies CFD,^1^ but the mechanics that dictate if a fracture occurs are unclear.^2^ Due to the catastrophic impact of CFD injuries, and the lack of treatment options to reverse the effects of SCI, there is an urgent and unmet need to improve our ability to predict, and therefore mitigate against, CFD.^3^ This requires a complete understanding of the global and local mechanisms underlying CFD to develop reliable and repeatable impact models, which do not currently exist and are essential to the design of new safety devices with innovative preventative measures.^3^

Despite several decades of experimental cadaver work, there are no repeatable, dynamic cadaver models that produce CFD.^3^ Furthermore, CFD is still widely described clinically as a “distractive‐flexion” or “hyperflexion” injury, caused by a caudally‐directed impact to the anterior part of the head,^4^ despite evidence that flexion alone cannot cause CFD.^3^, ^5^ As highlighted in a recent review article,^3^ this concept likely persists because the majority of research concerning the mechanisms of cervical spine injury is published in engineering journals, rather than clinical journals, and perhaps also due to a lack of distinction between *global* injury vectors (i.e., applied to the entire head–neck), and those occurring *locally* at the injured spinal level. *Intervertebral* distractive‐flexion^6^ and anterior translation^7^ can produce CFD in short spine segments, but these motions do not result from *global* flexion of the head–neck.^3^, ^8^

In the limited reports of cadaver head–neck tests producing CFD, vertebral motions at the dislocating level have been *qualitatively* described as combined *flexion* and *anterior translation* ^3^ but the local and global kinematics leading up to, and at the point of, CFD have not been quantified. Furthermore, previous studies have only reported the *peak* axial load experienced by head–neck specimens during global vertical compression that produces CFD,^9^, ^10^ but qualitative observation suggests that peak load *precedes* the dislocation event, which occurs at a lower axial load following additional compression displacement.^10^ Quantitative kinematic and kinetic data at, and preceding, the instant of CFD are required for a complete understanding of the injury.

### 1.1 Study context

**FIGURE 1:**
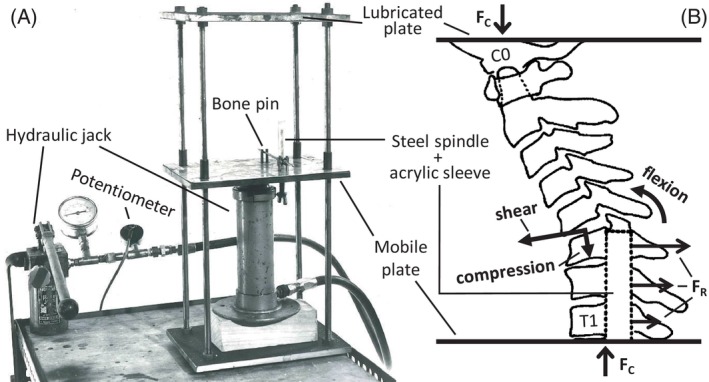
(A) Annotated photo of Bauze and Ardran's test setup; (B) schematic of a specimen in the “forward head position” during testing, with compression force (F_C_), reaction force (F_R_), and primary C5/C6 intervertebral motions (closed arrows; shear, compression, flexion) indicated.

In 1972/73, Adelaide‐based orthopedic surgeon Dr Robert Bauze, working at the Nuffield Department of Orthopedic Surgery, University of Oxford, was the first to systematically produce CFD in human cadaver necks.^10^ Fourteen fresh‐frozen cervical spines (Occiput‐T1), including the paraspinal muscles, all deeper tissues, and the caudal portion of the occiput (C0) with the C0/C1 joint maintained, were compressed between two horizontal plates (Figure 1). The under surface of the upper plate was lubricated so that C0 could translate in any direction as compression was applied. The caudal end of each specimen was fixed via a steel spindle placed in the spinal canal, and a bone pin through the most inferior vertebral body. Spindle height was adjusted relative to the anatomy in an attempt to influence the level of CFD. The occiput and upper cervical spine were “jammed” into extension, with a head‐forward (protracted) posture, at the start of each test. Additional vertical (axial) compression was applied to the specimens by raising the inferior plate using a hydraulic jack (Figure 1A). During loading, sagittal cineradiographs were captured on 35 mm film (4 Hz), and the output from a potentiometer in the hydraulic line (calibrated to the applied axial load) was recorded via a chart‐recorder (Mingograph), along with a trigger pulse to synchronize these data with the cineradiographs.

Bauze and Ardran reported subaxial CFD in 12 of 14 specimens, describing that vertical compression loading, and anterior translation of C0 (i.e., head protraction), caused extension of the upper cervical levels and intervertebral flexion and anterior translation at the level of CFD. This head–neck pose, analogous to “forward head posture,”^11^ was first described by Cornish in 1968 as comparable to the posture a person may adopt when “ducking in anticipation of a blow to the top of his head.”^12^ Decades later, Nightingale et al.^9^ reliably produced subaxial CFD by applying quasistatic axial compression to cadaver necks (C0–T1). With similar head‐end conditions to those of Bauze, Nightingale et al. observed that vertical compression produced the same forward head neck pose (they called “buckling”) and led to CFD. However, neither study reported *local* intervertebral kinematics (distraction, rotation, and/or shear motion) at the level of CFD, the *global* spinal motions prior to injury, or the axial force at the point of dislocation.

**FIGURE 2:**
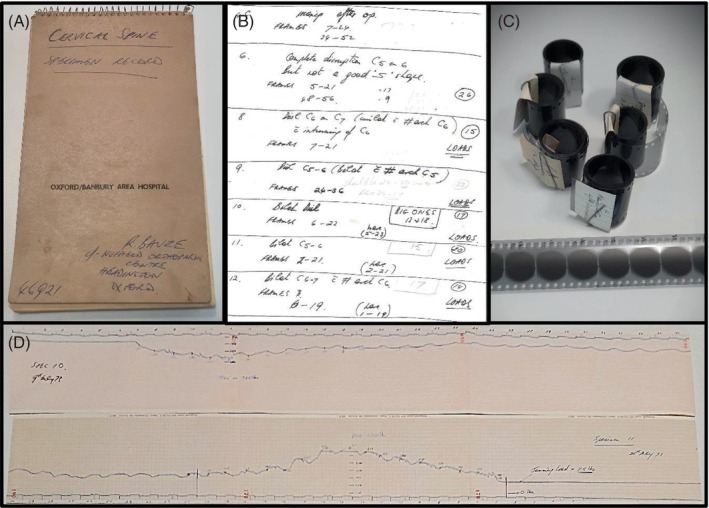
(A) Dr Robert Bauze's notebook; (B) hand‐written notes describing donor, specimen, and testing details; (C) 35 mm radiograph films; (D) chart‐recorder traces.

The importance of Bauze and Ardran's seminal work was highlighted in a recent review article,^3^ but their manuscript lacked quantitative kinematic and kinetic data, which is required to develop improved experimental and computer models of CFD. Fortunately, the original documentation from this experimental series was preserved (Figure 2). Therefore, the aim of this study was to re‐examine the analog data to quantify the local and global spinal motions, and axial forces, that occurred during the experiments that produced CFD.

## 2 METHODS

### 2.1 Material summary and record preservation

Research Ethics Committee approval was granted for this study (Approval No. H‐2020‐149). Dr Bauze's notebook provided additional donor, specimen, and testing details that were not included in the original manuscript. Fourteen cervical spines were tested, but iterative improvements to the apparatus and protocol meant that equivalent data were not collected for all specimens (Table 1). The sagittal cineradiographs of loading to injury were collected for 10 specimens (SP04–13), but one was omitted from kinematic analysis (SP05) because the cineradiograph did not capture the injury event (Table 1). The potentiometer was added to the hydraulic line from SP08 onwards, so chart‐recorder force data was available for six of these specimens. All available analog data items were digitized to preserve this historic dataset. The fragile cineradiograph films were digitally scanned (Atkins Photo Lab, Adelaide) and each frame was stored as an uncompressed JPEG image. The chart‐recorder traces were piecewise scanned, digitally stitched (GIMP 2.8.22), and exported as a single, high‐resolution bitmap image file (600 DPI).

**TABLE 1:** Donor demographics, specimen information, and data streams.

Specimen	Age	Gender	Cineradiographs?	Visible vertebra	Level of spindle	Force trace?	C7 VB width (mm)
SP01	67	M	**✗**	**✗**	**✗**	**✗**	**✗**
SP02	66	F	**✗**	**✗**	**✗**	**✗**	**✗**
SP03	31	F	**✗**	**✗**	**✗**	**✗**	**✗**
SP04	40	M	**✓**	C1–T1	C5/C6	**✗**	16.5
SP05	61	M	**✗**	C2–C7	C6	**✗**	**✗**
SP06	65	M	**✓**	C0–C7	C5/C6	**✗**	15.3
SP07	72	F	**✓**	C0–C7	C5/C6	**✗**	15.6
SP08	53	M	**✓**	C1–C7	C5/C6	**✓**	17.6
SP09	43	M	**✓**	C0–C6	C5	**✓**	**✗**
SP10	54	M	**✓**	C0–C7	C6	**✓**	16.7
SP11	60	M	**✓**	C0–T1	C6	**✓**	16.7
SP12	68	M	**✓**	C1–T1	C6	**✓**	16.3
SP13	67	M	**✓**	C0–T1	C6	**✓**	15.9
SP14	52	M	**✗**	**✗**	**✗**	**✗**	**✗**
Total or mean (SD)	57 (12)	11 M	9 **✓**	−	−	6 **✓**	16.3 (0.7)

Abbreviation: VB, vertebral body.

### 2.2 Cineradiograph analysis

**FIGURE 3:**
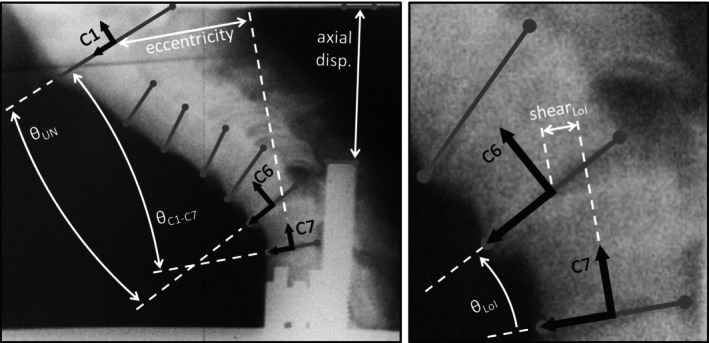
Global (left) and local (right) kinematic metrics obtained from each frame of digitized cineradiograph films. Gray lines represent the anteroposterior axis of each vertebra, defined by the anatomical landmarks (gray dots). Global specimen posture was the sagittal angle between C1 and C7 (θ_C1–C7_), and eccentricity was the anteroposterior distance between the midpoints of the vertebral bodies.^14^ Sagittal angle and shear translation were calculated at the level of injury (LoI), while upper neck angle was measured between C1 and the superior vertebra of the level of injury (θ_UN_). Average rate of axial displacement and the magnitude of axial compression of the specimen were determined from the position of the spindle. Distance between the C7 landmarks provided the C7 inferior endplate depth of each specimen.^15^

The analysis endpoint for each cineradiograph was defined by either: facet joint dislocation at any spinal level as characterized by a total absence of facet joint apposition^13^; or, complete separation of the occiput from the upper plate. Using custom MATLAB code (R2020a, MathWorks, MA, USA), the coordinates of two distinct landmarks were identified on each vertebra (C1–C7), the spindle, and the test rig frame, for each image (Figure 3). Although the specimens included C0 and T1, these levels were often obscured or out‐of‐frame, so distinct landmarks could not be reliably identified. For C1, the landmarks defined the anteroposterior axis, while the anterior and posterior corners of the inferior endplate were selected for C2–C7.^16^ The landmarks were used to build coordinate systems; local and global kinematics were calculated from linear transformation matrices derived between pairs of coordinate systems (Figure 3). Each set of images was calibrated (from pixels to millimeters) using test rig measurements documented in the notes.

### 2.3 Force trace analysis

**FIGURE 4:**
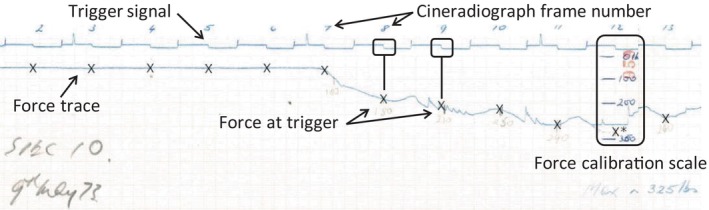
The scanned and stitched chart‐recorder trace for specimen SP10. × indicates the force value extracted from the force trace at each cineradiograph frame.

Chart‐recorder data were analyzed using MATLAB (Figure 4). Each image was calibrated (from pixels to Newtons) against the annotated scale using the custom function ‘GRABIT’ (v2.3).^17^ GRABIT was then used to measure the axial force at each trigger pulse (i.e., each frame of the cineradiography). The extracted kinematic and force data were synchronized, using the trigger pulses, to derive force‐displacement plots. Peak axial force, and force at CFD, were determined.

## 3 RESULTS

### 3.1 Materials summary

Of the nine experiments for which cineradiographs were analyzed, C0 was not clearly visible in three. Four specimens included T1, four ended at C7, and one ended at C6 (Table 1).

### 3.2 Features of observed injuries

Subaxial dislocation or fracture‐dislocation occurred in 8/9 experiments with cineradiographs (Table 2). The specimen that did not dislocate (SP09) fell away from the loading plate (via hyperflexion) due to a C5 vertebral arch fracture. CFD most often occurred at the C5/C6 (four specimens) and C6/C7 levels (three specimens), and one specimen dislocated at C4/C5 (Table 2). The level of dislocation corresponded with the level of the spindle for four specimens (all C5/C6), while vertebral arch fractures caused by the spindle led to dislocation below the level of fixation for two specimens. Concomitant C6 posterior element and C7 vertebral body fractures occurred for SP07, but no fractures were detected on the cineradiographs of the other five specimens that dislocated.

**TABLE 2:** Kinematic and kinetic outcomes, and injury details, for each specimen.

Specimen	Initial eccentricity (mm)	Loading rate (mm/s)	Displacement at injury (mm)	Final eccentricity (mm)	Change in LoI translation (mm)	Final C1–C7 angle (deg)	Final upper neck angle (deg)	Final LoI angle (deg)	Jamming force (N)	Peak force (N)	Failure force (N)	Injury	Primary mechanism
SP04	20.6	5.6	43.1	59.7	6.5	−28.5	41.3	−43.8	**✗**	**✗**	**✗**	C4/C5 dislocation	FHP
SP05	**✗**	**✗**	**✗**	**✗**	**✗**	**✗**	**✗**	**✗**	**✗**	**✗**	**✗**	None visible	N/A
SP06	25.1	10.1	20.4	54.7	1.1	−30.2	7.9	−26.3	**✗**	**✗**	**✗**	C5/C6 dislocation	FHP
SP07	39.5	10.7	19.1	60.1	4.0	−21.8	46.2	−40.5	**✗**	**✗**	**✗**	C5/C6 fracture dislocation	FHP
SP08	21.7	13.6	31.0	64.7	0.8	−37.3	−2.7	−34.6	303	861	460	C6/C7 fracture dislocation	FHP
SP09	**✗**	4.5	**✗**	**✗**	**✗**	**✗**	**✗**	**✗**	289	1300^a^	**✗**	None	N/A
SP10	19.5	8.8	20.4	42.2	3.7	−18.6	17.6	−34.8	234	1300^a^	684	C5/C6 dislocation	FHP
SP11	26.7	9.4	25.6	47.5	3.2	−7.4	38.3	−35.1	338	1300^a^	673	C5/C6 dislocation	FHP
SP12	16.0	9.4	20.7	41.2	4.0	−21.7	6.9	−28.6	289	917	570	C6/C7 fracture dislocation	FHP
SP13	47.5	10.1	30.0	78.2	1.5	−63.8	−29.2	−34.6	238	488	340	C6/C7 dislocation	Hyperflexion
Mean	27.1	9.1	26.3	56.0	3.1	−28.7	15.8	−34.8	282	755^b^	545		
SD	10.9	2.7	8.2	12.5	1.9	16.7	25.6	5.6	40	233^b^	147		

*Note*: Negative angles indicate flexion rotation.

Abbreviations: FHP, forward head posture; LoI, level of injury.

^a^ Force exceeded scale of chart‐recorder.

^b^ Excluding specimens that exceeded chart‐recorder limit.

The sequence of soft tissue and intervertebral injuries was consistent for all specimens in which CFD occurred, regardless of whether fracture occurred. Intervertebral flexion in the lower cervical spine caused rupture of the posterior ligaments. Compromise of these soft tissue structures allowed for additional intervertebral flexion rotation and shear translation, resulting in failure of the anterior longitudinal ligament and intervertebral disc, and subsequent CFD.

### 3.3 Kinematics

In the initial pose (“jamming position”^10^), which was achieved by gradually applying axial compression to the specimen until it was stable between the plates, specimens had an anterior eccentricity of 27.1 ± 10.9 mm (Table 2). Specimens were loaded until failure at between 4.5 and 13.6 mm/s. At failure, the specimens were compressed axially by 26.3 ± 8.2 mm, with an eccentricity (C1–C7) of 56.0 ± 12.5 mm, and an overall neck flexion angle (C1–C7) of 28.7 ± 16.7 degrees. At the level of injury, an intervertebral flexion angle of 34.8 ± 5.6 degrees and an increase in anterior shear translation (from initial pose) of 3.1 ± 1.9 mm caused CFD (Table 2).

**FIGURE 5:**
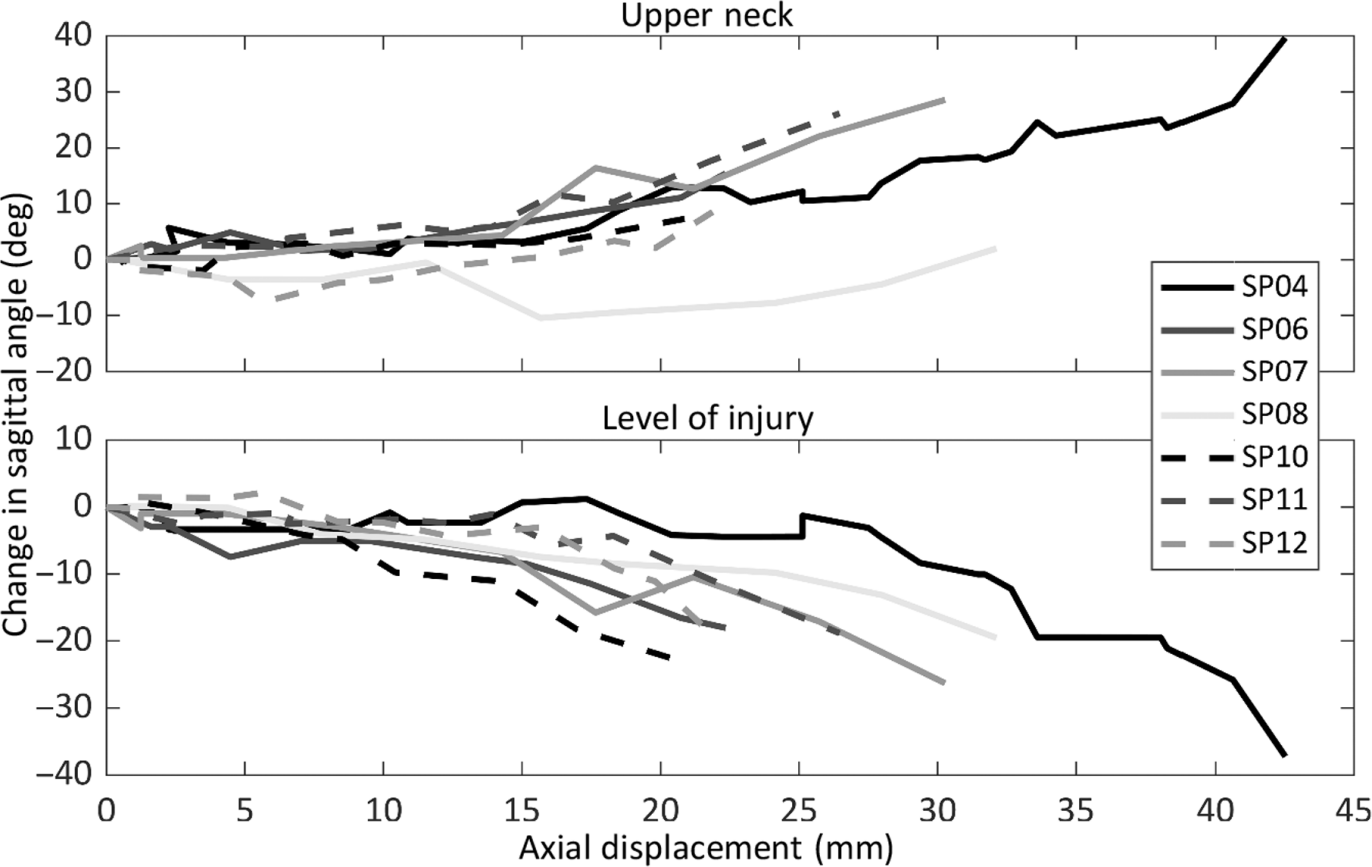
Change in sagittal angle (flexion negative) of the upper neck, and at the level of injury, versus axial displacement, relative to the start of loading, for each specimen that moved into the forward head posture.

**FIGURE 6:**
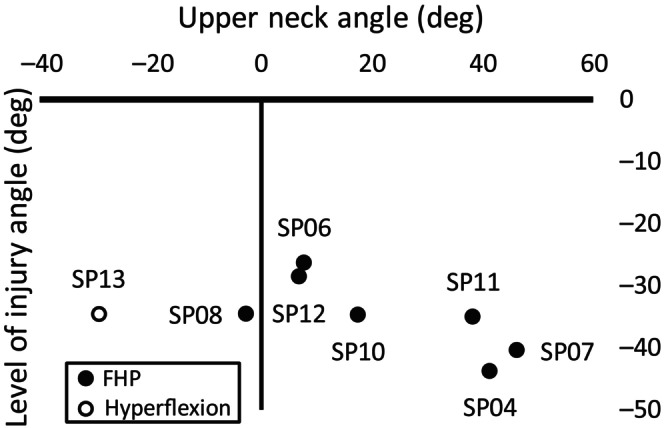
Sagittal angle at the level of injury versus upper neck angle, for each specimen. The mechanism of cervical facet dislocation is indicated by the color of each marker.

During loading, two distinct *global* spinal postures occurred to produce the *local* intervertebral motions preceding CFD. For seven specimens, a forward head posture occurred as axial compression was applied (Video S1 and Figure S3 and Figure 5). For these specimens, the upper neck alignment ranged from straight (SP08, 2.7 degrees of “flexion”) to >46 degrees extension (SP07), relative to the superior vertebra of the level of injury (Figure 6). In contrast, the anterior region of the occiput of one specimen (SP13) separated from the upper loading plate, resulting in *hyperflexion* of 29.2 degrees in the upper neck (C1–C6), and 63.8 degrees from C1 to C7 (Table 2).

### 3.4 Forces

**FIGURE 7:**
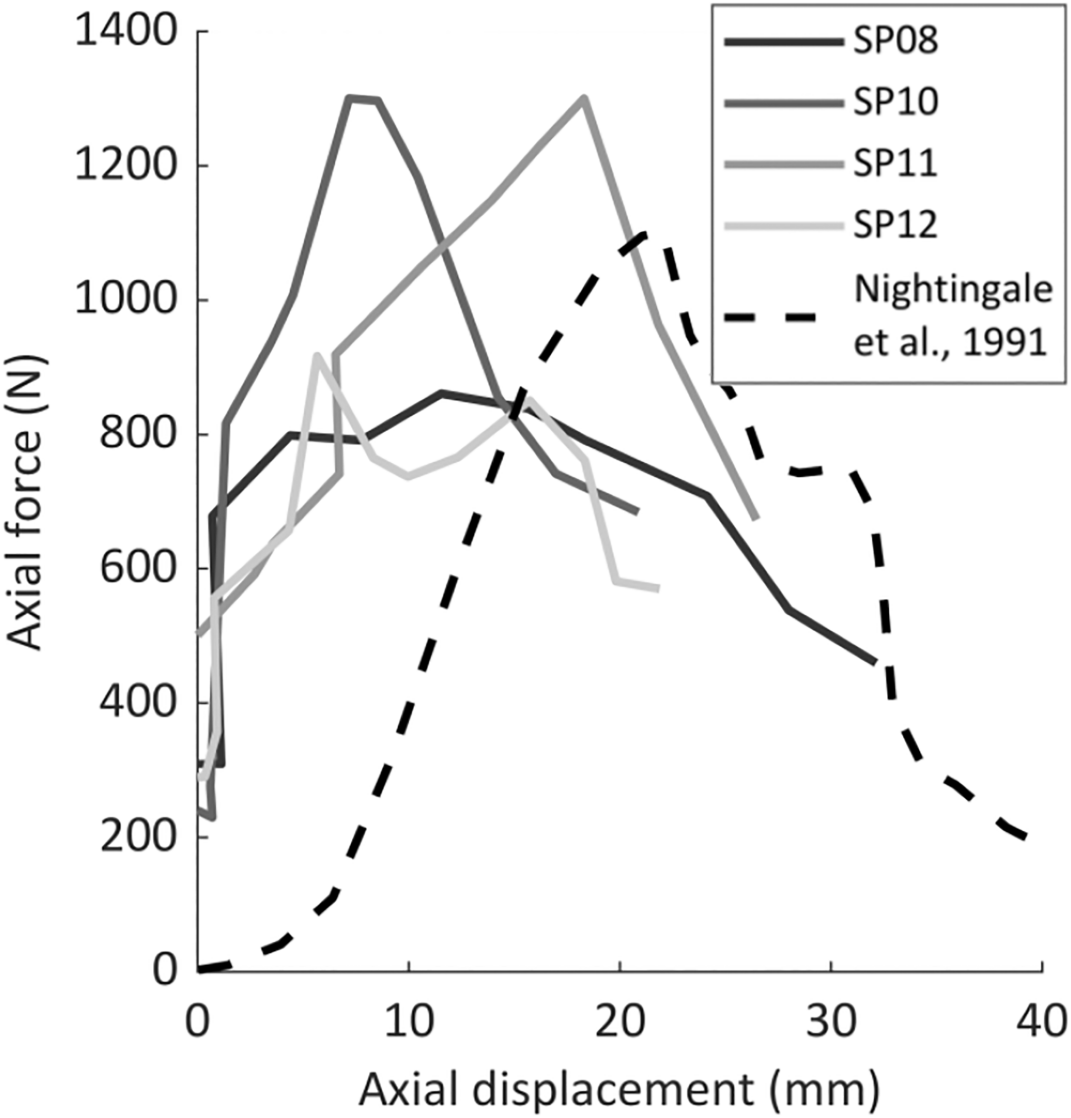
Axial force versus displacement plots from the four experiments with force strip charts that resulted in dislocation via the forward head posture. Equivalent data from Nightingale et al.^9^ is presented for comparison.

Of the six specimens with force data, four progressed to a forward head posture (*N* = 2 fracture‐CFD, *N* = 2 CFD without fracture), one had CFD via hyperflexion, and the last had no detected injury. The initial ‘jamming position’ corresponded to an axial force of 282 ± 40 N (Table 2). During three experiments the applied force exceeded the chart‐recorder limit (~1300 N), so peak force was not captured. However, the force corresponding to injury was captured for all specimens that dislocated. Peak force (755 ± 233 N, *N* = 4) appeared to coincide with fracture or failure of the posterior ligaments, and preceded dislocation (Figure 7).

## 4 DISCUSSION

Understanding of the injury mechanisms of CFD has been hindered by the lack of a reliable experimental model of the injury, and studies that have produced CFD have reported limited kinetic and kinematic data.^9^, ^10^ In this study, the original materials from the first reported human cadaver experiments to produce CFDs were re‐examined to investigate the local and global kinematics, and axial forces, that occurred at the point of dislocation. These data demonstrate two potential mechanisms of CFD arising from axial compression of the head and neck.

Seven specimens adopted a forward head posture as axial compression was applied, leading to CFD (Video S1). This posture produced intervertebral anterior translation (3.2 ± 2.1 mm) and flexion rotation (33.9 ± 6.1 degrees) exceeding physiologic limits,^7^, ^18^ with upper neck *extension* of 18.2 ± 17.9 degrees (Figure 6). The same posture was observed by Nightingale et al.^9^ during their quasi‐static experiments that produced CFD. Their reported axial displacement (15–39 mm), peak force (600–3590 N), and eccentricity (69.6 mm; from exemplar plot) leading to CFD were similar to the present study (Table 3). Axial force‐displacement responses during CFD in the current study were similar to the exemplar data presented by Nightingale et al.^9^ (Figure 7), but they did not report whether the peak force (which corresponded to posterior soft‐tissue failure in the present study) preceded, or was coincident with, CFD.

**TABLE 3:** Outcomes from the experimental studies that reported the kinematics and kinetics associated with cervical facet dislocation (CFD).

Study	No. of CFDs	Loading rate	Peak force (*N*)	Force at injury (*N*)	Displacement at injury (mm)	Final eccentricity (mm)	Upper neck/head pose at injury	Change in LoI translation (mm)	LoI flexion (deg)
Bauze and Ardran^10^	8	Quasi‐static (4.5–13.6 mm/s)	755.3 ± 233.1	596.9 ± 104.8	26.3 ± 8.2	56.0 ± 12.5	Extension	3.1 ± 1.9	34.8 ± 5.6
Nightingale et al.^9^	6	Quasi‐static (~20 mm/s)	1718.3 ± 1233.6	‐	29.2 ± 8.9	69.6	Extension	‐	‐
Nightingale et al.^8^, ^19^ (N18‐R + 15)	1	Dynamic (3.26 m/s)	2502.6	‐	‐	‐	Extension	‐	‐
Nightingale et al.^3^, ^19^ (N03‐P + 0)^a^	1	Dynamic (3.08 m/s)	3956.5	2052.8	30	‐	Extension	6.8	15.5
Ivancic^20^	1	Dynamic (2.4 m/s)	763.1 ± 149.1	‐	61 ± 29	79 ± 15	Extension	‐	‐

Abbreviation: LoI, level of injury.

^a^ Kinematic and kinetic data acquired from data presented in Nightingale et al.^3^

In addition to the quasistatic studies,^9^, ^10^ the forward head posture has also been described in the few studies that have produced CFD during dynamic axial loading of unconstrained head–neck specimens (Table 3). Nightingale et al.^8^, ^19^ produced C6/C7 bilateral CFD in 2/22 specimens, and attributed it to ‘buckling’ of the neck, causing extension of the head and upper neck, and flexion in the lower cervical spine (anterior head translation not reported).^3^, ^8^, ^19^ The sagittal high speed video for one of these specimens (N03‐P + 0) was provided in a 2019 review article.^3^ Kinematic analysis showed that neck axial deformation at CFD was similar to that in the current study and in Nightingale et al.^9^; however, at the injury level, shear was approximately double, and flexion half of those measured in the current investigation (described in Supplementary Material S1). This difference in local kinematics could be due to the unrestricted anterior translation of the head‐end in the quasistatic experiments, resulting in a local injury vector with a greater component of distractive‐flexion compared with when the head does not translate.

In the dynamic experiments, peak axial neck load and axial force at injury were greater than those reported during quasistatic loading that led to CFD in the current study, and by Nightingale et al.^9^ This difference in kinetics is most likely due to the inertia of the following torso mass. This is supported by a study that produced a CFD (in 1/10 head–neck specimens)^20^ by applying axial loading (2.4 m/s) with large anterior head eccentricity, such that the following torso mass did not compress the spine. Although specimen‐specific outcomes were not provided, the mean peak force amongst all specimens (all of which sustained posterior ligament failure) was comparable to the peak force observed in this study (Table 3).

The sequence of soft tissue and intervertebral injuries observed in the current study, and the associated kinematics and kinetics, were consistent with dynamic experimental CFD. Peak axial force corresponded to rupture of the posterior ligaments (Figure 7), followed by local hyperflexion (Figure 5), supraphysiologic shear (Table 2), and subsequent subaxial dislocation, without the head and upper neck exceeding physiological flexion.^3^, ^19^, ^20^ The force corresponding to a CFD in this pose (597 ± 105 N) was 83%–87% lower than the axial force associated with vertebral compression fractures produced in the laboratory,^3^, ^9^, ^19^ and only 15% of the compression limit (4000 N^21^) for the neck injury criteria, *N*_*ij*_. These data may help inform improved neck injury criteria which are sensitive to CFD.

In this study, hyperflexion appeared to cause CFD in one specimen. During axial loading of SP13, the anterior aspect of C0 fell away from the upper plate, causing the upper neck to flex and producing a C6/C7 dislocation (Table 2). However, the C1‐C7 flexion angle at the point of CFD (63.8 degrees) exceeded the anatomical chin‐to‐chest limit (40–55 degrees),^3^, ^9^, ^20^, ^22^, ^23^ indicating that this mechanism of CFD is unlikely in vivo.

In the original manuscript, Bauze and Ardran^10^ reported that dislocation occurred in 13/14 specimens. Interrogation of the testing notes revealed that the occiput did not translate for one specimen (SP01), producing a compression fracture of the C4 vertebral body instead of CFD. Injury mechanism could not be verified for four specimens because cineradiographs were not available (SP02, SP03, SP14) or did not capture the injury event (SP05). Cineradiograph analysis of the other nine experiments confirmed that the described injuries occurred during axial loading for all except SP09. During this experiment, the C5 vertebral arch fractured through the spindle causing C0 to disconnect from the loading plate and the specimen to fall over (via hyperflexion) without an associated CFD. However, as additional compression was applied to the posterior aspects of C0‐C2, a C5/C6 dislocation injury was produced. Although Bauze and Ardran reported this CFD, it did not occur during axial loading, so all data after C0 lost contact with the loading plate was excluded from analysis in the current investigation.

Unlike the subsequent experimental studies that produced CFD, concomitant fractures of the posterior elements occurred immediately prior to, or during, dislocation for three specimens (Table 2). Axial load at CFD was similar for those with a fracture, compared with those without (566 ± 196 vs. 515 ± 78 N, respectively), but these fractures almost certainly occurred due to the spindle, which likely acted as a non‐physiologic stress concentrator on the lamina and may have altered the injury mechanics. It should also be highlighted that although the injury to SP07 has been classified as a C5/C6 fracture‐dislocation (Table 2), the ‘dislocation’ occurred because of a shearing fracture through the C6 vertebral body, representing a different injury mechanism to the other specimens.

The limitations of this analog dataset need to be considered when interpreting the outcomes. To determine loading rate, axial displacement, and intervertebral motions, images were calibrated to documented test rig dimensions. Inaccuracies in calibration could occur due to out‐of‐plane errors and optical distortion. However, the C7 vertebral body dimensions obtained from the calibrated images (Table 1) are comparable to those measured directly in anthropometric studies of human cadaver cervical spines.^15^

The variable quality of the cineradiographs (inconsistent focus, motion blur, and contrast) prevented implementation of automatic and semi‐automatic tracking algorithms, so landmarks were identified manually. Kinematic measurement accuracy was also limited by out‐of‐plane vertebral motions which could not be accounted for on the sagittal cineradiographs.

The measurement techniques employed by Bauze and Ardran were innovative for the time, but some of the inherent limitations of the work could not be overcome in this contemporary analysis of the data. The applied axial compression force during each experiment was measured using a pressure transducer (“potentiometer,” Figure 1A) in the hydraulic fluid line, which was pre‐calibrated by placing known masses on the mobile plate attached to the jack. No other loads were measured, so the shear force and flexion moment associated with CFD could not be determined. In addition, peak axial force could not be determined for three specimens with force traces as the applied force exceeded the chart‐recorder range. Therefore, the peak force associated with a CFD was only available for two specimens (SP08 and SP12) that dislocated below the spindle top following C6 lamina fracture, likely due to the spindle acting as a stress‐concentrator. Despite this, the peak axial forces were similar to that reported for quasistatic and dynamic CFD production.^9^, ^20^

The experimental model of cervical spine injury developed by Bauze and Ardran,^10^ and later elegantly replicated by Nightingale et al.,^9^ is the only model to repeatedly produce subaxial CFD in head–neck cadaver specimens, and has provided invaluable insight into the mechanisms underlying the injury. Bauze and Ardran were the first to describe the sequence of posterior soft‐tissue injury preceding dislocation, and to demonstrate that axial compression can produce intervertebral shear and compression in the lower cervical spine, leading to CFD *without* head–neck hyperflexion. However, the loading and boundary conditions implemented are likely not representative of those that occur during a head impact that causes CFD. Axial compression was applied at quasistatic rates (mean 9.1 ± 2.7 mm/s) in a step‐wise manner via a manually operated hydraulic jack, but a loading rate of 3 m/s is generally thought to be the minimum required to produce spinal column injuries during head impacts.^8^, ^24^, ^25^ To facilitate the neck pose leading to CFD (i.e., the “forward head posture”), occiput translation was permitted (mean 29.0 ± 8.4 mm) but rotation was constrained; however, inverted drop tests of head–necks have demonstrated that cervical spine injuries precede any motion of the head following impact.^26^ This likely indicates that for subaxial CFD to occur during dynamic axial compression, the head–neck must already be adopting a forward head posture at the onset of neck loading.

At the caudal end, the lower spinal canal was mounted onto a steel spindle, attached to the mobile plate, that was fitted with a specimen‐specific acrylic sleeve. Bauze and Ardran^10^ hypothesized that CFD would occur at the spinal level immediately superior to the location of fixation. To test this, the length of the spindle within the canal was varied between specimens (Table 1). CFD only occurred at the level adjacent to the spindle for four of the seven specimens that adopted a forward head posture (Table 2). As discussed above, in two specimens the spindle likely acted as a non‐physiologic stress concentrator and caused vertebral arch fractures, leading to dislocation at the level of the spindle, superior to the fixed level(s) that did not fracture. For the other specimen (SP04), the top of the spindle was placed at the superior endplate of C6 (i.e., intended to induce CFD at C5/C6), but dislocation occurred at C4/C5. Kinematic analysis showed little intervertebral sagittal rotation at C5/C6 (<8°) throughout this experiment and inspection of the cineradiographs revealed reduced disc height at this level, suggesting degeneration and increased intervertebral stiffness.^27^ Although this method of fixation is unphysiological and likely influenced failure mechanics, the re‐examination performed in this study has provided new evidence that supports Bauze and Ardran's^10^ hypothesis that CFD occurs at the level of transition from the relatively stiff lower cervical spine to the more mobile middle and upper cervical spine.

The mechanics of CFD are of contemporary interest in the injury biomechanics and clinical research communities.^3^, ^28^, ^29^ Re‐examining this rich dataset has provided new quantitative evidence supporting the hypothesis that head–neck posture and axial compression are risk factors for facet dislocation. It is anticipated that this information will assist with the development of improved neck injury criteria that can predict CFD, and with the design of advanced injury prevention devices. Digital copies of all the original materials have been made available online at Figshare.

## AUTHOR CONTRIBUTIONS

*Conceptualization*: RDQ, RB, and CFJ. *Methodology*: RDQ, RB, and CFJ; *Validation*: RDQ, RB, and CFJ. Formal analysis: RDQ; *Investigation*: RDQ, RB, and CFJ. *Resources*: RDQ, RB, and CFJ. *Data curation*: RDQ. *Writing—original draft*: RDQ and CFJ. *Writing—review and editing*: RDQ, RB, and CFJ. *Visualization*: RDQ. *Supervision*: RB and CFJ. *Project administration*: RDQ and CFJ. *Funding acquisition*: RDQ and CFJ.

## CONFLICT OF INTEREST STATEMENT

The authors declare no conflicts of interest.

## Supporting information


**Data S1** Supporting Information.
