# Supplementary material for: Kinematics, kinetics, and new insights from a contemporary analysis of the first experiments to produce cervical facet dislocations in the laboratory
Source: JOR Spine. 2024 May 27;7(2):e1336. doi: 10.1002/jsp2.1336 (PMC11129552; doi:10.1002/jsp2.1336)
Supplement: Supplementary file 1 — Data S1 Supporting Information. [file JSP2-7-e1336-s001.docx]

# SUPPLEMENTARY MATERIAL A: NIGHTINGALE KINEMATIC AND KINETIC DATA ANALYSIS

Important kinematic and kinetic data describing cervical facet dislocation were extracted from existing datasets in the literature.

## High speed video analysis

Local kinematics were determined from the high speed video footage of experiment N03-P+0, which produced a C6/C7 bilateral facet dislocation during Nightingale *et al.*’s head-impact testing series [1, 2]. Intervertebral shear translation and flexion rotation angle were calculated by tracking the markers on the vertebral body and spinous process of C6 and C7 using the ‘Video Labeler’ MATLAB application (**Fig. S1-A**). Shear translation measurements were converted from pixels to millimeters by calibrating against the compressive displacement values specified in Nightingale *et al.*, 2019 [3] (**Fig. S1-B**).


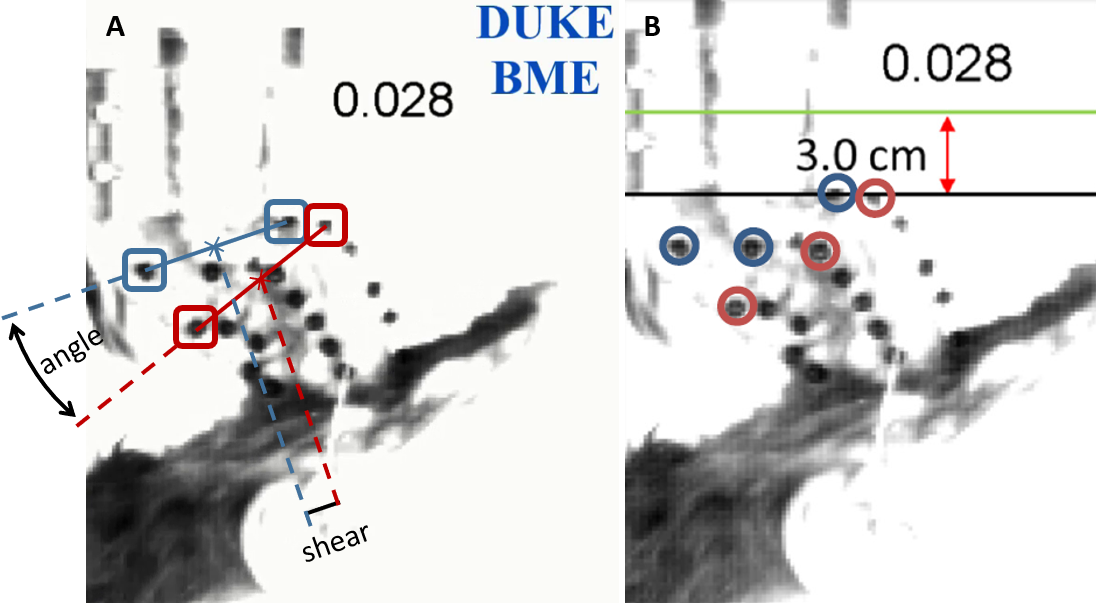


**Fig. S1** A) Intervertebral anteroposterior shear and sagittal angle at the level of cervical facet dislocation (C6/C7) were measured from the high speed video for N03-P+0 [1, 3]. The red and blue solid lines represent the anteroposterior axes of C6 and C7, respectively, defined by the markers on each vertebral body and spinous process. B) Axial compression at injury was previously reported [3], and this measurement was used to convert the shear measurements from pixels to mm

## Force trace analysis

Using the custom MATLAB function ‘GRABIT’ (v2.3) [4], axial force data was extracted from the caudal-end load cell traces reported for the two experiments that produced bilateral cervical facet dislocation in Nightingale *et al.*’s head-impact testing series (**Fig. S2**). Peak axial force, and force at injury, were determined for N03-P+0. Only peak load could be determined for N18-R+15, as the timepoint corresponding to injury was not indicated.


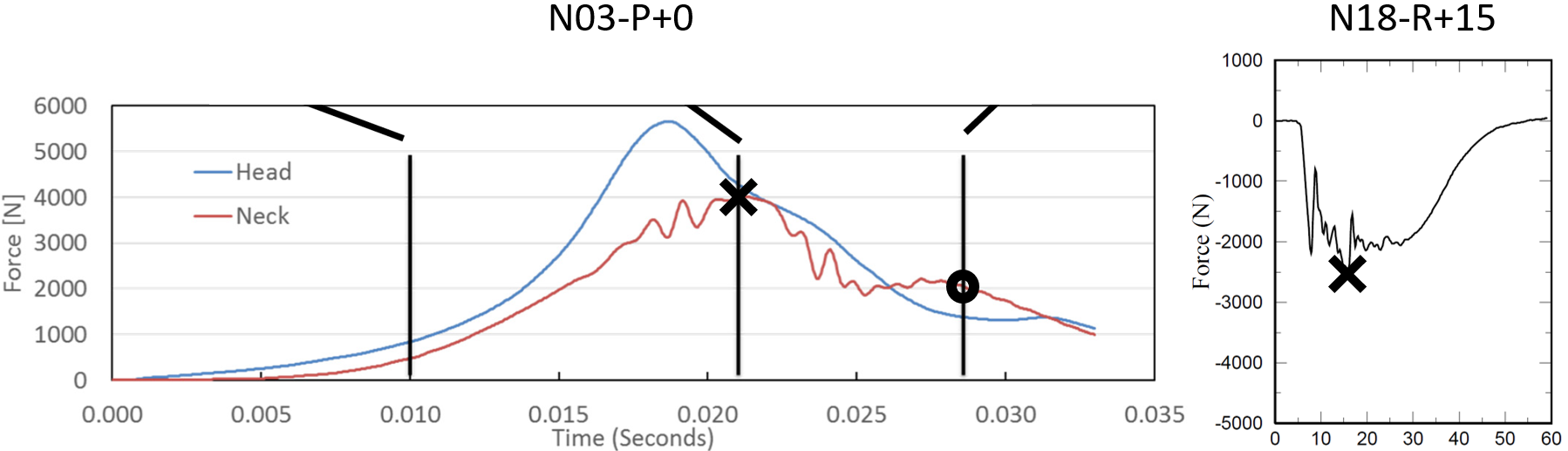


**Fig. S2** Peak axial neck force (🞬) and force at injury (🞇; N18-R+15 only) were extracted from the traces provided in Nightingale et al. 2019 [3] (left) and Nightingale et al. 1997 [1] (right)

## References

1. Nightingale RW, McElhaney JH, Camacho DL, Kleinberger M, Winkelstein BA, Myers BS (1997) The dynamic responses of the cervical spine: buckling, end conditions, and tolerance in compressive impacts. SAE transactions:3968-3988

2. Nightingale RW, McElhaney JH, Richardson WJ, Myers BS (1996) Dynamic responses of the head and cervical spine to axial impact loading. Journal of biomechanics 29:307-318

3. Nightingale RW, Bass CR, Myers BS (2019) On the relative importance of bending and compression in cervical spine bilateral facet dislocation. Clin Biomech 64:90-97. doi: 10.1016/j.clinbiomech.2018.02.015

4. Doke J (2021). GRABIT. https://www.mathworks.com/matlabcentral/fileexchange/7173-grabit, MATLAB Central File Exchange. Retrieved June 8, 2021.

# SUPPLEMENTARY MATERIAL B: STILL IMAGES FROM SP11 CINERADIOGRAPH VIDEO

The digitised still images from the original cineradiograph films for SP11 are presented in Fig S3. Onset of loading occurred at Frame 6 and CFD occurred at Frame 16.


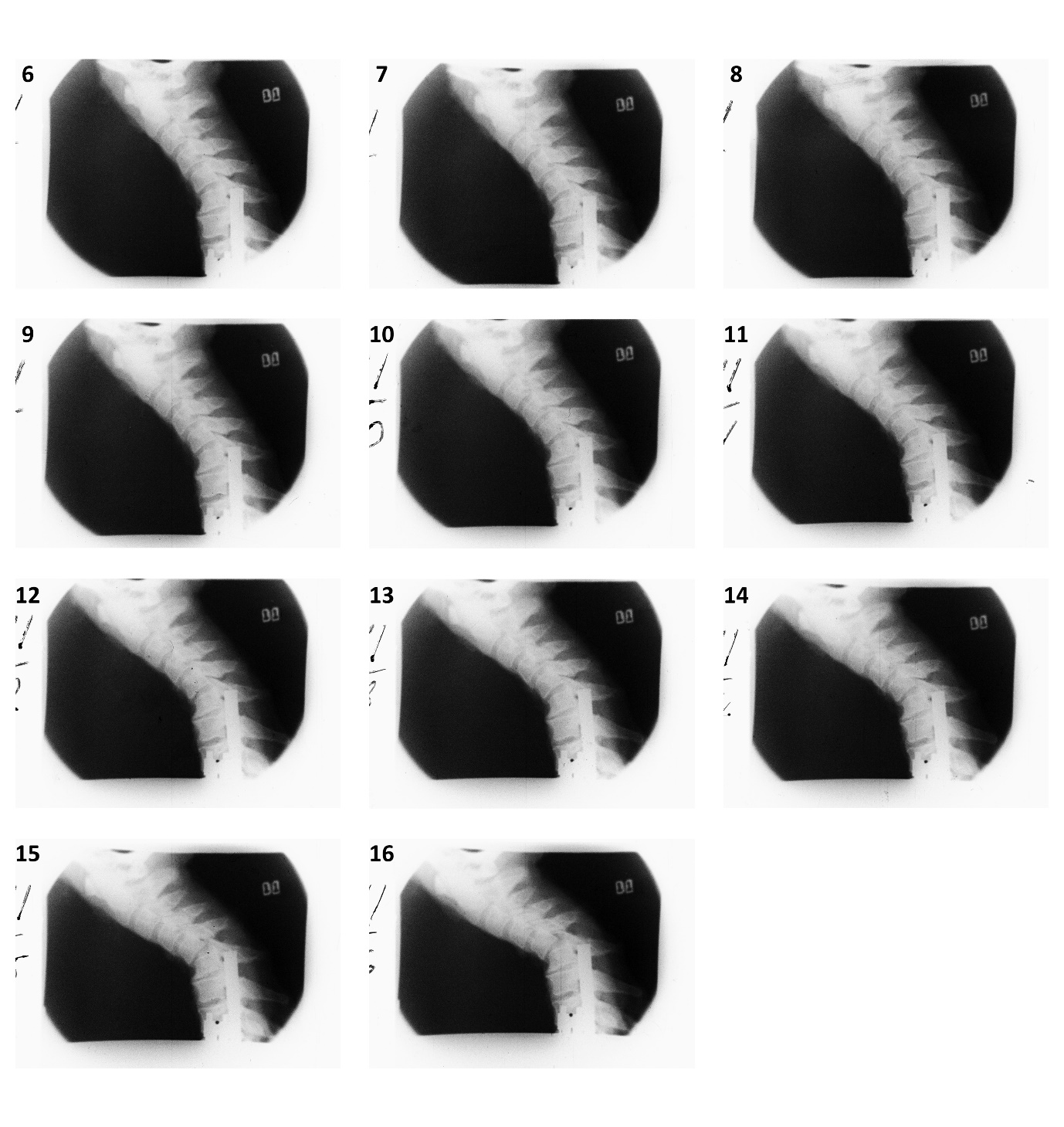


**Fig. S3** The digitised still images from the cineradiograph of testing SP11.
